# Integrating static and modifiable risk factors in violence risk assessment for forensic psychiatric patients: a feasibility study of FoVOx

**DOI:** 10.1080/08039488.2022.2084158

**Published:** 2022-06-13

**Authors:** Jonas Forsman, Robert Cornish, Seena Fazel

**Affiliations:** a Department of Clinical Neuroscience, Karolinska Institutet, Stockholm, Sweden; b The Oxford Clinic, Littlemore Hospital, Oxford Health NHS Foundation Trust, Oxford, UK; c Department of Psychiatry, University of Oxford, Oxford, UK

**Keywords:** Forensic psychiatry, prognosis, risk assessment, feasibility, FoVOx

## Abstract

**Introduction:**

Risk assessment is integral to forensic psychiatry. Previous work has highlighted the benefits of using scalable and evidence-based actuarial risk tools developed within forensic populations, such as the online Forensic Psychiatry and Violence Oxford (FoVOx) violence risk assessment tool. We examined the feasibility of using FoVOx in a Swedish forensic cohort and tested whether adding modifiable (dynamic) factors would increase its useability to clinicians.

**Methods:**

We completed FoVOx assessments on all patients discharged from forensic psychiatric hospitals in Stockholm County, Sweden, between 2012 and 2017 and investigated recidivism rates. In addition, interviews were conducted with the clinicians responsible for each patient on the perceived accuracy, usefulness, and impact of FoVOx, which was examined using thematic analysis.

**Results:**

Ninety-five discharges from forensic psychiatric hospitals were followed up. The median FoVOx score was a 7% likelihood of violent reoffending in two years after discharge. Six discharged patients (6%) were confirmed as violent recidivists using official records with a similar distribution of FoVOx risk categories as the rest of the sample. FoVOx was considered accurate by clinicians in more than half of cases, who suggested that modifiable risk factors could be added to increase acceptability. All clinicians thought that FoVOx was useful, and in 20% of discharges, it would have materially altered patient care. Overall, FoVOx was thought to impact decision-making and risk management, was practical to use, and could be completed without reference to written case material.

**Conclusion:**

Completing FoVOx in forensic psychiatric hospitals can complement current approaches to clinical decision-making on violence risk assessment and management.

## Introduction

Risk assessment is an integral part of forensic psychiatric practice. The process of gatekeeping new patients into a secure hospital setting, or readmitting them, is generally dependent on an assessment of the seriousness of their risks to others [1]. Many interventions in secure psychiatric hospital focus on decreasing the likelihood of causing future harm, and a reduction in risk is central to discharge planning, often as a criterion under mental health law or related legislation. Despite the importance of accurate risk assessment, there are challenges in how it is currently conducted. In clinical settings, structured professional judgement tools are generally preferred to actuarial assessments of risk. Although there are benefits, in that individual risk formulation can be constructed, drawbacks have been noted by experts [2]. These include poor field validity [3,4], containing items which are not predictive [5] leading to redundancy and waste, and their implementation and use in populations different to those in which they were developed. Further, they are often time consuming to complete, and do not provide an easily interpretable quantified assessment of risk [6].

Therefore, there has been increasing discussion in the use of evidence-based actuarial tools [7] developed specifically for forensic psychiatric populations, and that are scalable, transparently developed, and validated. These can improve the accuracy of risk assessment without adding significantly to the burden on staff. Doing so may also increase the time available for risk management and violence prevention, rather than purely focusing on assessment. One such tool, the Forensic Psychiatry and Violence tool Oxford (FoVOx), has demonstrated good performance in terms of discrimination (a tool’s ability to distinguish between those who have the outcome of interest and those who do not by assigning risk score or category to those with the outcome) with a reported AUC of 0.77, and sensitivity (true positive rate) of 55% and specificity (true negative rate) of 83% using a 20% probability score as the cut off for elevated risk of violent reoffending [8]. The FoVOx tool has also demonstrated good calibration (how well the tool’s predicted risk matches with the actual observed risk), which has not been reported in previous risk assessment instruments but a key performance metric [9]. The FoVOx tool was developed using multivariate models and, unlike other tools, was based on an adequate sample size for tool development. FoVOx has, furthermore, also been internally validated, and the coefficients and formula for its output have been published.

In this investigation, we aimed to investigate the FoVOx tool in a Swedish forensic psychiatric setting, with a focus on feasibility and pilot validation data. This is the first such study in a Nordic country. Secondary aims were to examine how it could implemented and developed, including monitoring risk.

## Materials and methods

### Study design and participants

We used a mixed-method approach to investigate the feasibility of FoVOx and examined data on its predictive performance by: (i) identifying discharged forensic psychiatric patients in the Swedish National Forensic Psychiatric Register (RättspsyK); (ii) scoring their risk using the FoVOx tool; (iii) qualitatively assessing the tool by interviews with the clinicians in charge at discharge and; (iv) conducting a pilot investigation of the recidivism rates in the patient cohort based on their FoVOx score.

### Setting

The Swedish National Forensic Psychiatric Register (RättspsyK) [10] is a national quality register which has collected a range of socio-demographic, criminal history and clinical data on patients sentenced to forensic psychiatric care since 2008. Twenty-four (out of 25) Swedish forensic psychiatric units annually report to this register with a current national patient coverage of around 85%. In addition to basic patient information (age, sex, geography, date of admission and discharge), the register contains and annually collects data on 25 indicators, including ICD-based psychiatric and somatic diagnoses, types of treatment, level of care and accommodation. The Swedish National Crime Register provides data on all crime convictions in Sweden in individuals aged 15 and over (the age of criminal responsibility) since 1973.

### Patients

All patients registered in RättspsyK and discharged from forensic psychiatric care in Stockholm County to the Swedish community between 1 January 2012, and 31 December 2017, were identified and included in the study cohort.

### Clinicians

All lead clinicians (consultant level or equivalent) for the patient cohort at the time of discharge were identified and contacted for interview. This comprised seven women and seven men, all specialist psychiatrists but not all sub-specialized in forensic psychiatry.

### Measures

#### FoVOx

Information to calculate each included patient’s FoVOx score was extracted from RättspsyK. FoVOx is an online violence risk assessment tool that consists of twelve items, including socio-demographic, criminal history, and clinical factors, which are mostly categorized dichotomously. When there was missing data, such as status of employment prior to conviction, as in previous work [11], necessary information was reliably completed from available health records. The Swedish translated online version FoVOx (available at https://oxrisk.com/fovox-7/) was used to calculate risk scores (a probability of violent offending at 1 and 2 years after discharge that ranges from 0 to 60%, with the highest score set at a ceiling of >60%) and to present FoVOx to clinicians during interviews.

#### Questionnaire

A Swedish version of a previously developed semi-structured feasibility questionnaire was used to interview clinicians (Supplementary Appendix 1–2). Each clinician went through an in-depth interview with a combination of predetermined options and open-ended questions regarding each of their assessments prior to discharge. So that clinicians could familiarise themselves with their patient prior to the interview, they were asked to read an extract of their own previous psychiatric report for the court (which is completed every 6 months in Sweden). The standardized questionnaire contained no patient identifiable information.

As part of this interview, the clinician was asked to estimate at discharge the two-year risk of a violent conviction in terms of the pre-specified FoVOx categories (Low <5%; Medium 5–20%; High >20%). In instances of a given overlapping risk range (e.g. low-medium, or medium-high), the highest risk was recorded. The clinician was then asked if they knew whether the patient had committed a violent offence since discharge.

After this, the clinician was informed of the calculated FoVOx risk assessment score and risk category of their patient at discharge. The clinician’s view and reasoning, as well as thoughts of FoVOx potential use at previous discharge, were then recorded. In each instance, the clinician was asked to provide reasons of why FoVOx would or would not have altered the previous clinical management. Lastly, a verbal summary of the collected information was given at the end of each interview for the clinician to confirm or specify further. The records of the open-ended questions were individually analysed and thematically organized by two interviewers, who are both specialist psychiatrists (JF, HB). In a follow-up consensus meeting, principal themes were finally identified and decided in accordance with previous work and newly found categories.

### Pilot validation of FoVOx

Each included patient was identified in the National Crime Registry regarding sentenced violent crime convictions in Sweden in accordance with previous definitions [8]. Specified dates of when the crimes were committed were used to calculate time periods from discharge to violent re-offence. A cut-off of 730 days was used to validate the performance of FoVOx two-year risk prediction post discharge.

### Ethics

The research ethics committee in Stockholm, Sweden approved the research project (reference number 2019-04048). To identify patients, existing data on discharges in the RättspsyK was used. No patient data beyond what had been collected through routine clinical care or previous informed consent as part of inclusion in the RättspsyK was used. Management of patients or registry data was not impacted by the study. All interviewed clinicians participated in the study voluntarily under informed consent, and patient data was anonymized other than for the ‘unblinding’ during the interviews.

## Results

### Sample

**Figure 1.**
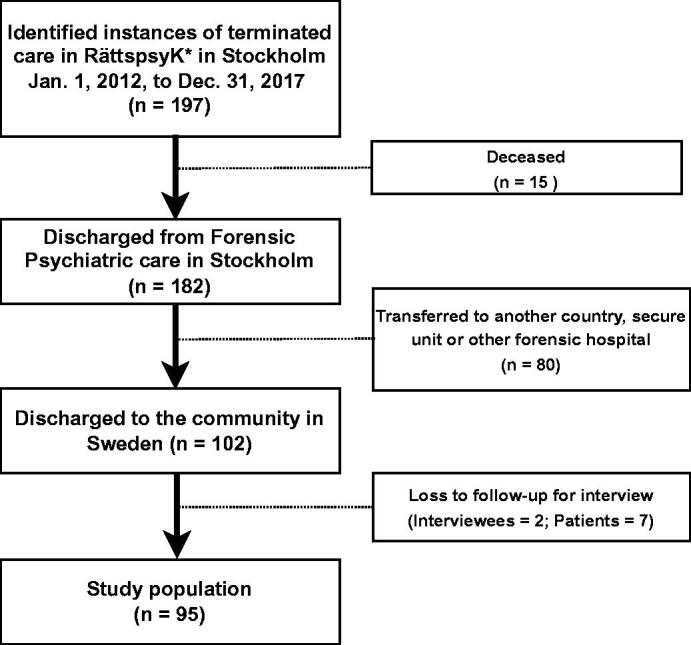
Flow chart of discharged forensic psychiatric patients that were included in the study.

A total of 197 discharges from forensic psychiatric care in Stockholm County were identified from 1 January 2012 to 31 December 2017. Ninety-five patients were not included in the follow-up (15 were registered as having died from any cause and 80 patients had been transferred to another country, secure unit, or other forensic psychiatric hospital). An additional seven patients were excluded due to loss to follow-up (as two clinicians in charge of their care that did not participate for interviews out of a total of 14 consultant psychiatrists in charge). Therefore, 95 patients discharged to the community in Sweden were included in the study (Figure 1). Of these, 15 (16%) were female and the median age was 46 (range 21–82).

The number of assessed patients per clinician ranged from 1 to 25 and the median time from discharge to study interview was 2141 days (interquartile range 1788–2602 days). Eight out of 12 (67%) clinicians reported the use of a structured risk assessment tool in addition to clinical interviews at the time of discharge. These were the Short-Term Assessment of Risk and treatability (START) (*n* = 2) or HCR-20 (*n* = 3), or a combination of both (*n* = 2). One clinician reported the use of Violence Risk Appraisal Guide (VRAG).

### Baseline characteristics

Sample characteristics and distribution of FoVOx-specific risk factors are presented in Table 1. Of the sample, 89 (94%) had previously been sentenced for a violent crime, 88 (93%) had over one year of current inpatient stay, and 91 (91%) had at the time of their detention been unemployed for at least six months. 44 (46%) of the cohort had previously committed a serious violent crime and 39 (41%) had a history of drug abuse. The most common primary diagnosis at discharge was schizophrenia spectrum disorder (*n* = 46, 48%). In those that had new violent convictions after discharge, median age at discharge was 36. In comparison to the full study sample, those who had violently reoffended were less likely to be male or have schizophrenia spectrum disorder and multiple previous inpatient episodes. All other risk factors were more common among those committing violent crimes after discharge.

**Table 1.** Baseline characteristics.

Variables	All patients (*n* = 95)		Violent recidivists, convicted (*n* = 6)	
Age, median (range)	46 (21–82)		36 (30–59)	
Age, mean (SD)	47 (15)		41 (11)	
	*n*	%	*n*	%
Sex, male	80	84	3	50
Previous violent crime	89	94	6	100
Previous serious violent crime	39	41	5	83
Primary discharge diagnosis				
* Schizophrenia spectrum*	*46*	*48*	*1*	*17*
* Bipolar disorder*	*5*	*5*	*0*	*0*
* Unipolar depression*	*0*	*0*	*0*	*0*
* Anxiety disorder*	*1*	*1*	*0*	*0*
* Other*	*43*	*45*	*5*	*83*
Drug use disorder at hospitalization or discharge	30	32	4	67
Any previous drug use disorder (lifetime)	44	46	5	83
Alcohol use disorder at hospitalization or discharge	35	37	3	50
Personality disorder diagnosis at discharge	7	7	3	50
Up to 6 months before admission, which can be full/part-time education	9	9	1	17
Five or more previous psychiatric inpatient episodes	24	25	1	17
Length of current inpatient stay >1 year	88	93	6	100

### FoVOx scores

FoVOx scores were calculated prior to the interviews for each patient from RättspsyK data and clinical records. The median FoVOx probability score for violent reoffending within two years was 7% (range 0% to 40%) for the overall sample. Regarding FoVOx pre-specified risk categories, 28 (30%) were estimated to be low risk, 60 (63%) medium risk, and 7 (7%) high risk.

### Recidivism

Of the 95 discharges, 9 (9%) were reported to have committed further violent offences based on the information from the clinician in charge. Of these, five patients had FoVOx scores in the medium category and one in the high category. Two of these, and four other patients (*n* = 6, 6%) were identified in the crime register to have been convicted for new violent crimes within two years after discharge. Among the convicted violent recidivists, five were categorized as medium or high risk.

### Concordance between FoVOx scores and clinical judgment

Dichotomizing the risk assessment (*low* versus *medium/high*), the agreement between clinician and FoVOx scores was 47% (42 out of 90, kappa = 0.09 [95% CI, −0.05–0.24].) The clinician’s versus FoVOx risk ratings are presented in Table 2.

**Table 2.** Risk categories assigned by clinicians versus categories based on FoVOx scores.

	All patients (*n* = 95)	Violent recidivists, convicted (*n* = 6)
FoVOx categories		
Low	28 (30%)	1 (17%)
Medium	60 (63%)	4 (67%)
High	7 (7%)	1 (17%)
Clinician’s assessment at discharge		
Low	64 (67%)	2 (33%)
Medium	21 (22%)	4 (67%)
High	5 (5%)	0 (0%)
Don’t know	5 (5%)	0 (0%)

	FoVOx (all patients)		
Clinicians (all patients)	Low	Medium	High
Low	20	36	5
Medium	6	15	2
High	0	6	0
	Low	Medium/High	
Low	20	41	
Medium/High	6	23	

FoVOx risk categories are based on pre-specified risk levels. Low: < 5%; medium: 5–20%; high:> 20%.

In most cases (*n* = 60, 63%), the clinician in charge considered the FoVOx risk assessment to be an accurate representation of the actual risk of violence at discharge. In 24 (25%) instances, clinicians did not think FoVOx accurately reflected this risk. Identified reasons as to why FoVOx was not an accurate representation of the risk were mostly based on the relative proportion of modifiable (dynamic) and static factors in the tool, and whether FoVOx was considered to overestimate or underestimate risk (Table 3.) Missing modifiable factors in either direction were thought to be ‘*level of insight’* and ‘*recurrent and compulsive thoughts*’. Some protective factors that clinicians considered that a high FoVOx did not consider were: ‘*an uncomplicated patient*’; ‘*stability and progress of given care*’; and ‘*well-coordinated social support measures*’. Other relevant factors were: ‘*relapse of substance abuse’*; ‘*impulsivity*’; ‘*oddness of index crime’*; and ‘*adherence to medication*’. Among static (non-modifiable) factors that were considered missing when FoVOx was considered to overestimate risk were ‘*severe somatic illness*’, *‘misjudged primary diagnosis at discharge*’ and ‘*honor-related violence*’. Low risk FoVOx assessments were in a few instances thought to miss possible static risk factors such as ‘*dementia’, ‘psychopathy/manipulative behavior’*, and ‘*autism’*. ‘*Level of accommodation*’ was repeatedly mentioned as both a static risk factor and as a protective factor against future violence.

**Table 3.** Qualitative feedback on additional factors not considered in FoVOx.

	General	Factors reducing risk	Factors increasing risk
Modifiable Risk Factors	Risk of relapse of substance abuse Varied adherence to medication Other individual factors	Gained insight Clinical presentation of patient over time Good adherence to medication Ceased recurrent and compulsive thoughts Well coordinated social measures	Lack of insight Ongoing recurrent and compulsive thoughts Specific types of index violence
Static Risk Factors	Oddness of index crime Other individual factors	Severe somatic illness Suitable accommodation Inaccurate diagnosis at discharge Honor-related violence	Dementia Psychopathy/Chronic manipulative tendencies Not arranged accommodation Autism not specifically taken into account Impulsivity

### Viewpoints on utility at the point of discharge

All the interviewed clinicians expressed that FoVOx would have been of clinical benefit at the time of discharge. Additionally, in 20 (20%) discharges, clinicians thought that the instrument would have materially altered their assessment and management. The qualitative feedback is summarized in Table 4. In instances when FoVOx was helpful, clinicians stated that it: ‘*corresponded and supported our clinical judgment*’; ‘*would have added an additional objective argument*’; ‘*the results would have been easy to communicate with the court, community, and patient*’; and ‘*would have highlighted the overall risk in a more specific way than just the overall clinical judgment*’. Comments that considered FoVOx not helpful were: *‘assessment would have been based on other factors, including modifiable factors’* and ‘*a general clinical impression is of greater value than specific risk points’.*

**Table 4.** Psychiatrists’ qualitative feedback on FoVOx future utility.

Themes	Sub-themes	Helpful	Potential limitations
Assist decision-making	As part of the discharge planning	Speeds the decision-making when the tool corresponds to the subjective risk assessment.	Other factors had a much higher impact than the FoVOx factors in the discharge planning.
	In communication with third parties	Simple and easy to use for communication with the court, relatives and the community.	Risk assessment tools cannot solely be referred to in the Swedish court system.
	In communication with the patient	Gives the patient an objective argument of why he/she has a certain risk level.	Lacks dynamic factors, which patients often ask for over the course of several assessments.
Impact of risk management	Early perception of risk	Gives a quick objective view of the risk for this specific type of patient.	The lack of dynamic or other factors might give false assurance or hesitancy.
	Planning patient management	Highlights risk factors previously not considered by junior colleagues or other health care workers.	May give false assurance or hesitancy.
	Reassurance or control	Similar risk assessment of FoVOx strengthens the clinician’s confidence and choice of action.	General clinical impression of greater value than actuarial risk points.

### Overall views of practicality and future use

In terms of practical use, all clinicians found the FoVOx-web-based tool to be practical, and the majority (*n* = 8, 67%) reported that the tool could be completed without referring to clinical notes. Nine clinicians (or 75%) planned to use FoVOx in the future, whereas two clinicians were unable to say, and one would not use the tool referencing current work with non-forensic psychiatric patients. Reasons against the use of FoVOx were: ‘*not suitable for every patient*’ and ‘*it might give a false risk assessment when specific variables are not covered*’. Common reasons for future use included that FoVOx is: ‘*possible to use both in regard to termination and continued care*’; ‘*it’s made simple to compare risk factors*’; ‘*it will be very useful for junior colleagues and other specialties*’; and ‘*it is very relevant*, *easy to use, well-structured and time-efficient.*’

## Discussion

From the Swedish National Forensic Psychiatric Register (or RättspsyK), we identified and completed individual FoVOx risk assessments on 95 discharged forensic psychiatric patients in Stockholm County. We then interviewed 12 specialist psychiatrists who were lead clinicians at the time of patient discharge. These interviews assessed previous risk assessments by these clinicians, useability and usefulness of FoVOx, perceived accuracy, and potential improvements. Lastly, we investigated the sample’s probability scores of violent offending after discharge from hospital based on the tool and compared these with officially recorded convicted violent crimes as part of a pilot external validation.

In keeping with previous studies [12–14], clinicians found the FoVOx tool easy and practical to use, as well as reliable. Despite mixed concordance between FoVOx probability scores and the clinical judgments at time of discharge, most clinicians nevertheless considered that FoVOx presented an accurate representation of the risk of violent reoffending. The calculated median risk (7%) of violent reoffending within two years post discharge was consistent with officially recorded convictions for violent crimes (6%) over two years, but lower than the median risk (11%) of the target population (all discharged forensic psychiatric patients in Sweden during 1992 to 2013) from which FoVOx was developed. The extent to which the tool captured the unexplained variance of violence reoffending was not directly tested but the Brier score, which is a measure of calibration or the extent of the correspondence between expected and observed outcome rates, provides one approach and was tested in the FoVOx development sample. The Brier score can range between 0 and 1 and quantifies the accuracy of a tool's risk prediction by averaging the squared differences between the predicted and observed outcome probabilities [15]. Based on the internal validation, the tool performed very well for the two main outcomes at 24 months (Brier score 0.09) and 12 months (0.06), where 0 would be a perfect score and 1 would be poor [8,16].

In the qualitative analysis, consistent with previous feasibility studies, some clinician impressions were that FoVOx lacked modifiable and some specific static risk factors. Clinicians suggested missing static factors included oddness of the index offence, statutory supervision at the point of discharge, discharge to supported accommodation, other specific chronic diagnoses, and chronicity of past violence. Further work could investigate whether adding these additional factors could incrementally improve FoVOx accuracy. In relation to ‘oddness of the index crime’, although such offences have been studied and incorporated in criminal personality profiling since the 1970s [17] and associated with some cases of autism spectrum disorder and psychosis [18], it has not to date been integrated as a static item in any violence risk instrument. Clinician respondents were generally more focused on adding modifiable factors, which is understandable given the need to provide interventions to reduce the risk of violent recidivism [19]. Based on this and previous FoVOx feasibility studies, possible modifiable risk factors could include: current substance abuse, adherence and response to medication, impulsivity, recency of violence post-sentence (any recorded interpersonal violence on the inpatient ward, home or community after their index sentence date), insight, and psychosocial support and employment after discharge. This is also consistent with qualitative work about risk assessment more generally in forensic settings [20–25]. However, one risk factor that has not been identified in qualitative work but reported in the current study is ‘recency of violence post-sentence’. Some of these clinical factors are contained in other risk assessment tools, such as FoxWeb [26] which is based around 10 modifiable factors, and has been recently validated [27]. As with FoVOx, FoxWeb is quick to complete, includes predictors that are reliably coded, and requires little training. Since it focuses only on modifiable predictors, the use of FoVOX and FoxWeb together would address clinician concerns about actuarial tools and enable risk monitoring over time. Future work could assess the feasibility of using two separate tools (or combining them) including testing whether the inclusion of new factors would incrementally improve the performance of FoVOx, and its acceptability among clinicians. Previous work has noted that adding certain clinical factors, such as the poor adherence, and psychosocial factors, such as community supervision, may increase the tool’s acceptability to clinicians [12,14]. Apart from FoxWeb, the Structured Outcome Assessment and Community Risk Monitoring (SORM) was an attempt to continuously measure around 30 modifiable factors among forensic psychiatric patients and developed in Sweden. However, it has not maintained clinical use, possibly due to its complexity [28] and lack of ongoing advocacy. Other work [29] that has used Bayesian networks for risk assessment has yet to be tested and externally validated among forensic patients, and may also be too complicated for translation into practice. 

A central aim of working with forensic psychiatric patients is to reduce the risk of recidivism. One of the benefits of using tools such as FoVOx is that its brevity and ease of use frees up more time for risk management. In the future, trials could examine whether the implementation of scalable risk assessment tools improves outcomes, and whether incorporating the strategies identified above, such as improving adherence with treatment and facilitating meaningful daytime activity, prevents reoffending on discharge from secure hospital. Further work could investigate the role of more regular follow-up by clinical services or multidisciplinary review, enhancing medication adherence by optimising antipsychotic treatment and considering intramuscular administration [30], and offering psychological therapies to address substance misuse and other comorbidities [31]. This may involve closer liaison between forensic and general adult community mental health services, along with substance misuse treatment providers to provide timely intervention.

## Limitations

One limitation is that any comparison of clinical judgement with risk assessment tools using thresholds depends on what clinicians understand that the categories low, medium and high mean. It will also need to consider that a statutory requirement for termination of forensic psychiatric care under special court supervision in Sweden is that there should not be any remaining risk of repeat offending of a serious nature, including violence against the person. In practice, this means that all discharged persons will be deemed low risk by clinical teams, and the FoVOx threshold of <5% may not reflect what clinicians mean by low risk. In contrast, most of the sample had medium risk scores (5–20% probability of repeat violent offending within 2 years), and if a threshold of <20% was used, then the concordance between FoVOx and clinical rating would have been nearly perfect. This discrepancy may also explain the variation between the risk of recidivism in our sample (median 7%) compared to the original sample from which the FoVOx tool was developed (11%), as only the lower risk cohort can actually be discharged (although caution is warranted in this interpretation as the numbers were small). Those posing a higher risk of recidivism will have remained in hospital. This variance may also be accounted by subtle changes in practice over time, and a move towards risk averseness.

The number of repeat offenders in this pilot was small, and not sufficient for an external validation. Further research is warranted, including a larger updated validation of the Swedish forensic psychiatric rates of violent recidivism. In addition, this study only examined violent reoffending but multiple adverse outcomes should be considered on discharge. In particular, high rates of mortality have been reported in forensic patients [32].

In conclusion, in this first feasibility study of Fovox in a Nordic country, we found using mixed-methods that the tool was acceptable, easy to use, positively impacted on decision-making, and could be used as a complement to current clinically-led approaches. The incremental utility of adding more modifiable factors is an area for future research.

## Supplementary Material

Supplemental Material

Supplemental Material

## Data Availability

All data is subject to ethical approval, and cannot be shared with third parties.

## References

[1] Fazel S, Wolf A, Fimińska Z, et al. Mortality, rehospitalisation and violent crime in forensic psychiatric patients discharged from hospital: rates and risk factors. PLoS One. 2016;11(5):e0155906–14. 27196309 10.1371/journal.pone.0155906 PMC4873227

[2] Silva E. The HCR-20 and violence risk assessment – will a peak of inflated expectations turn to a trough of disillusionment? BJPsych Bull. 2020;44(6):269–271. 33213557 10.1192/bjb.2020.14 PMC7684770

[3] Vojt G, Thomson LDG, Marshall LA. The predictive validity of the HCR-20 following clinical implementation: Does it work in practice? J Forensic Psychiatry Psychol. 2013;24(3):371–385.

[4] Jeandarme I, Pouls C, De Laender J, et al. Field validity of the HCR-20 in forensic medium security units in Flanders. Psychol Crime Law. 2017;23(4):305–322.

[5] Coid JW, Yang M, Ullrich S, et al. Most items in structured risk assessment instruments do not predict violence. J Forensic Psychiatry Psychol. 2011;22(1):3–21.

[6] Singh JP, Fazel S, Gueorguieva R, et al. Rates of violence in patients classified as high risk by structured risk assessment instruments. Br J Psychiatry. 2014;204(3):180–187. 24590974 10.1192/bjp.bp.113.131938 PMC3939440

[7] Hvidhjelm J, Sestoft D, Skovgaard LT, et al. Sensitivity and specificity of the Broset violence checklist as predictor of violence in forensic psychiatry. Nord J Psychiatry. 2014;68(8):536–542. 24506491 10.3109/08039488.2014.880942

[8] Wolf A, Fanshawe TR, Sariaslan A, et al. Prediction of violent crime on discharge from secure psychiatric hospitals: a clinical prediction rule (FoVOx). Eur Psychiatry. 2018;47:88–93. 29161680 10.1016/j.eurpsy.2017.07.011 PMC5797975

[9] Van Calster B, McLernon DJ, Van Smeden M, et al. Calibration: the achilles heel of predictive analytics. BMC Med. 2019;17(1):1–7. 31842878 10.1186/s12916-019-1466-7 PMC6912996

[10] RättspsyK. Swedish National Forensic Psychiatric Register 2020. Annual 2020. Gothenburg. 2020.

[11] Långström N, Grann M, Tengström A, et al. Extracting data in file-based forensic psychiatric research: some methodological considerations. Nord J Psychiatry. 1999;53(1):61–67.

[12] Zhong S, Yu R, Cornish R, et al. Assessment of violence risk in 440 psychiatric patients in China: examining the feasibility and acceptability of a novel and scalable approach (FoVOx). BMC Psychiatry. 2021;21(1):1–9. 33653305 10.1186/s12888-021-03115-3 PMC7923307

[13] Krebs J, Negatsch V, Berg C, et al. Applicability of two violence risk assessment tools in a psychiatric prison hospital population. Behav Sci Law. 2020;38(5):471–481. 32633430 10.1002/bsl.2474

[14] Cornish R, Lewis A, Parry OC, et al. A clinical feasibility study of the forensic psychiatry and violence oxford (FoVOx) tool. Front Psychiatry. 2019;10(December):1–8. 31920751 10.3389/fpsyt.2019.00901 PMC6928566

[15] Fazel S, Burghart M, Fanshawe T, et al. The predictive performance of criminal risk assessment tools used at sentencing: systematic review of validation studies. J Crim Justice. 2022;81:101902. 36530210 10.1016/j.jcrimjus.2022.101902 PMC9755051

[16] Brier GW. Verification of forecasts expressed in terms of probability. Mon Wea Rev. 1950;78(1):1–3.

[17] Wilson P, Lincoln R, Kocsis R, et al. Validity, utility and ethics of profiling for serial violent and sexual offenders. Psychiatr Psychol Law. 1997;4(1):1–11.

[18] Långström N, Grann M, Vladislav R, et al. Risk factors for violent offending in autism spectrum disorder: a national study of hospitalized individuals. J Interpers Violence. 2009;24(8):1358–1370. 18701743 10.1177/0886260508322195

[19] Tabita B, De Santi MG, Kjellin L. Criminal recidivism and mortality among patients discharged from a forensic medium secure hospital. Nord J Psychiatry. 2012;66(4):283–289. 22212020 10.3109/08039488.2011.644578

[20] Haggård-Grann U, Gumpert C. The violence relapse process – a qualitative analysis of high-risk situations and risk communication in mentally disordered offenders. Psychol Crime Law. 2005;11(2):199–222.

[21] Pollak C, Palmstierna T, Kald M, et al. It had only been a matter of time before I had relapsed into crime: aspects of care and personal recovery in forensic mental health. J Forensic Nurs. 2018;14(4):230–237. 30080709 10.1097/JFN.0000000000000210

[22] Askola R, Soininen P, Seppänen A, et al. Offense-related issues in forensic psychiatric treatment: a thematic analysis. Front Psychiatry. 2020;10(January):1–12. 10.3389/fpsyt.2019.00925 PMC6961555 31998150

[23] Olsson H, Audulv Å, Strand S, et al. Reducing or increasing violence in forensic care: a qualitative study of inpatient experiences. Arch Psychiatr Nurs. 2015;29(6):393–400. 26577553 10.1016/j.apnu.2015.06.009

[24] Radovic S, Höglund P. Explanations for violent behaviour - an interview study among forensic in-patients. Int J Law Psychiatry. 2014;37(2):142–148. 24314798 10.1016/j.ijlp.2013.11.011

[25] Berg J, Kaltiala-Heino R, Löyttyniemi V, et al. Staff’s perception of adolescent aggressive behaviour in four European forensic units: a qualitative interview study. Nord J Psychiatry. 2013;67(2):124–131. 22774936 10.3109/08039488.2012.697190

[26] Fazel S, Toynbee M, Ryland H, et al. Modifiable risk factors for inpatient violence in psychiatric hospital: prospective study and prediction model. Psychol Med. 2021;2021:1–7. 10.1017/S0033291721002063 PMC9899559 34024292

[27] Gulati G, Cornish R, Al-Taiar H, et al. Web-based violence risk monitoring tool in psychoses: pilot study in community forensic patients. J Forensic Psychol Pract. 2016;16(1):49–59. 26924945 10.1080/15228932.2016.1128301 PMC4743616

[28] Grann M, Sturidsson K, Haggård-Grann U, et al. Methodological development: structured outcome assessment and community risk monitoring (SORM). Int J Law Psychiatry. 2005;28(4):442–456. 16005968 10.1016/j.ijlp.2004.03.011

[29] Coid JW, Ullrich S, Kallis C, et al. Improving risk management for violence in mental health services: a multimethods approach. Programme Grants Appl Res. 2016;4(16):1–408. 27929618

[30] Sariaslan A, Leucht S, Zetterqvist J, et al. Associations between individual antipsychotics and the risk of arrests and convictions of violent and other crime: a nationwide within-individual study of 74 925 persons. Psychol Med. 2021;2021:1–9. 10.1017/S0033291721000556 PMC9811342 33691828

[31] Sariaslan A, Lichtenstein P, Larsson H, et al. Triggers for violent criminality in patients with psychotic disorders. JAMA Psychiatry. 2016;73(8):796–803. 27410165 10.1001/jamapsychiatry.2016.1349 PMC5047356

[32] Uhrskov Sørensen L, Bengtson S, Lund J, et al. Mortality among male forensic and non-forensic psychiatric patients: matched cohort study of rates, predictors and causes-of-death. Nord J Psychiatry. 2020;74(7):489–496. 32248726 10.1080/08039488.2020.1743753

