# Supplementary material for: Integrating static and modifiable risk factors in violence risk assessment for forensic psychiatric patients: a feasibility study of FoVOx
Source: Nord J Psychiatry. 2022 Jun 13;77(3):240–6. doi: 10.1080/08039488.2022.2084158 (PMC10108825; doi:10.1080/08039488.2022.2084158)
Supplement: Supplemental Material [file IPSC_A_2084158_SM0537.pdf]

Detta frågeformulär är en del av en studie kring möjligheten att använda FoVOx webbaserade skattningsformulär som ett våldsriskinstrument. Verktyget har utvecklats av Forensic Psychiatry and Psychology Group vid Oxford University, som en del av projektet OxRisk.

FoVOx-verktyget är utformat för att ge stöd vid våldsriskbedömningar av rättspsykiatriskt dömda individer.

Frågeformuläret riktar sig till ansvariga kliniska riskbedömare som skattar risken för återfall i allvarlig brottslighet i samband med ansökan om övergång till annan vårdform hos rättspsykiatriskt dömda individer. Syftet med formuläret är att undersöka huruvida ansvariga riskbedömare anser formuläret användbart vid framtida riskbedömningar samt reflektera över tidigare bedömningar.

Unikt ID-nummer/personnummer av den individ som bedömts: ________________________________

Datum för ändrad vårdform: ________________________________

Typ av ändrad vårdform:
□ Övergång från sluten till öppen rättspsykiatrisk vård
□ Avslut av den rättspsykiatriska vården

Q1. Vid den ändrade vårdformen, hur uppskattade du individens risk för utövande av fysiskt eller sexuellt våld mot andra?

□ **Låg** (<5% risk för någon form av övergrepp inom två år från utskrivningen)
□ **Medel** (5-20% risk för någon form av övergrepp inom två år från utskrivningen)
□ **Hög** (>20% risk för någon form av övergrepp inom två år från utskrivningen)
□ **Minns inte**

Q2. Såvitt känt, har individen begått något våldsbrott sedan vårdformen ändrades?

□ **Ja**
□ **Nej**
□ **Minns inte**
□ **Kontakt med individen har upphört**

Här kommer forskaren att informera riskbedömaren om individens beräknade FoVOx-skattning vid tiden för den ändrade vårdformen.

Observera att de frågor som hör samman med en * endast kommer att ställas till riskbedömaren vid ett tillfälle.

Q3. Är din uppfattning att FoVOx-skattningen återspeglade den faktiska risken?

□ **Ja**
□ **Nej**
□ **Vet inte**

Q3b – Om inte, varför?

____
____

Q4a. Tror du att kännedom om FoVOx-skattningen skulle ha medfört någon fördel? Exempelvis skulle skattningen ha förändrat din tidigare riskbedömning?

□ **Ja**
□ **Nej**

Q4b. Varför?/Hur?

____
____
____
____

Q5a*. Använder du rutinmässigt något våldsriskinstrument i dina rättspsykiatriska riskbedömningar?

□ **Ja**
□ **Nej**

Q5b*. Om ja, vilket/vilka instrument använder du dig av?

____
____

Här visar forskaren riskbedömaren en utskrift / skärmbild av FoVOx och den information som krävs för att genomföra en skattning.

Observera att de frågor som hör samman med en * endast kommer att ställas till riskbedömaren vid ett tillfälle.

Q6a*. Efter att ha sett FoVOx-instrumentet, tror du att dess webbaserade formulär skulle vara praktisk att använda i rollen som riskbedömare vid ansökan till förvaltningsrätten om ändrad vårdform, inklusive avslut av den rättspsykiatriska vården?

□ **Ja**
□ **Nej**

Q6b*. Om inte, varför då?

____

____

Q7*. Tror du att det i det flesta fall skulle vara möjligt att genomföra en FoVOx-skattning utan något som helst stöd av anteckningar/journalhandlingar?

□ **Ja** □ **Nej**

Q7a*. Kommer du att använda dig av FoVOx i framtiden?

□ **Ja**
□ **Nej**

Q7b*. Varför, eller varför inte?

____

____

____

Q8*. Har du några andra kommentarer kring FoVOx?

____

____

____

____

____

____

____

____

____

____

Observera att de frågor som hör samman med en * endast kommer att ställas till riskbedömaren vid ett tillfälle.
