# Supplementary material for: Integrating static and modifiable risk factors in violence risk assessment for forensic psychiatric patients: a feasibility study of FoVOx
Source: Nord J Psychiatry. 2022 Jun 13;77(3):240–6. doi: 10.1080/08039488.2022.2084158 (PMC10108825; doi:10.1080/08039488.2022.2084158)
Supplement: Supplemental Material [file IPSC_A_2084158_SM0536.pdf]

OXRISK

This questionnaire is part of a study into the feasibility of using the FoVOx web-based risk calculator. This tool was developed by the Forensic Psychiatry and Psychology group at the University of Oxford, who, as part of a project called OxRisk, develop freely available web-based risk calculators.

The FoVOx tool is designed to assist in assessing the risk of violence in a forensic psychiatry-based population.

This questionnaire is aimed at Responsible Clinicians assessing risk of violence in forensic inpatients at the point of discharge. The objective of the questionnaire is to discover whether Responsible Clinicians would find the FoVOx tool acceptable to use in clinical practise.

The discharge number of this case is: ____

The date of discharge of this patient is: ____

(The patient's name and discharge location would also be identified by the researcher at this point)

Q1. Regarding the patient discharge episode identified above: at the point of discharge, what was your estimate of the patient's risk of physical or sexual violence to others?

□ **Low** (<5% risk of offending within 2 years of discharge)
□ **Medium** (5-20% risk of offending within 2 years of discharge)
□ **High** (20%+ risk of offending within 2 years of discharge)
□ **Unable to recall**

Q2. To your knowledge, has the patient committed a violent offence since discharge?

□ **Yes**
□ **No**
□ **Unable to recall**
□ **Patient lost to follow up**

At this point the researcher will inform the clinician of the patient's FoVOx score at the point of discharge.

Q3. Do you think that the FoVOx score was an accurate representation of the risk that this patient posed at discharge?

□ **Yes**
□ **No**
□ **Unable to say**

Q3b – If no, then why not?

____

____

Note that all questions associated with a * will only be asked on one occasion to each responsible clinician.

Q4a. Do you think that knowing the patient's FoVOx score at the point of discharge would have been of any clinical benefit e.g would it have altered your management of this patient?

□ **Yes**
□ **No**

Q4b. Why/how?

____
____
____
____

Q5a*. Do you routinely use a risk assessment tool or process at discharge?

□ **Yes**
□ **No**

Q5b*. If yes, what tool or process do you use?

____
____

At this point the researcher shows the clinician a printout/screen shot of the tool and the information required to achieve a risk assessment.

Q6a*. Having seen the tool, do you think that the FoVOx web-based calculator would be practical to use as part of a discharge assessment/plan?

□ **Yes**
□ **No**

Q6b*. If no, why not?

____
____

Q7*. Do you think that it would be possible to complete the FoVOx tool WITHOUT having to refer to the clinical notes in the majority of cases?
□ **Yes** □ **No**

Q7a*. Will you use FoVOx in the future?

□ **Yes**
□ **No**

Q7b*. Why or why not?

____
____
____

Q8*. Do you have any other comments you wish to make regarding the tool?

____
____

Note that all questions associated with a * will only be asked on one occasion to each responsible clinician.
